# H2Bub1: Guardian of chromatin accessibility in ovarian cancer

**DOI:** 10.18632/oncoscience.484

**Published:** 2019-08-23

**Authors:** Hunter Reavis, Ronny Drapkin

**Affiliations:** Ovarian Cancer Research Center, Department of Obstetrics and Gynecology, University of Pennsylvania Perelman School of Medicine, Philadelphia, PA, USA; Basser Center for BRCA, Abramson Cancer Center, University of Pennsylvania School of Medicine, Philadelphia, PA, USA

**Keywords:** Fallopian tube, ovarian cancer, RNF20, H2Bub1, chromatin

In 2019, an estimated 22,530 women will be diagnosed with ovarian cancer in the United States. Because patients with localized ovarian cancer are typically asymptomatic, greater than 60% of newly diagnosed patients present with advanced, metastatic disease for which there are no curative therapies [1]. One of the most challenging aspects of this disease is early diagnosis. Recent studies aim to better understand the pathogenic origins of ovarian cancer in order to identify biomarkers and therapeutic strategies to target oncogenic drivers during early disease development.

Mounting evidence supports the notion that high-grade serous ovarian carcinomas (HGSOCs) arise from fallopian tube (FT) epithelial precursors [2,3,4]. Gene expression patterns of HGSOCs most closely resemble those of the matched FT epithelium, as opposed to ovarian surface epithelium or peritoneum [2]. FT cells that acquire *TP53* mutations are thought to accumulate further genomic instability that can eventually progress to form serous tubal intra-epithelial carcinomas (STICs) that precede HGSOC development. In addition to the common genetic features shared by precursor FT cells and HGSOC, recent methylome analyses have linked early DNA methylation changes to preserved patterns in advanced disease [5,6,7].

**Figure 1:**
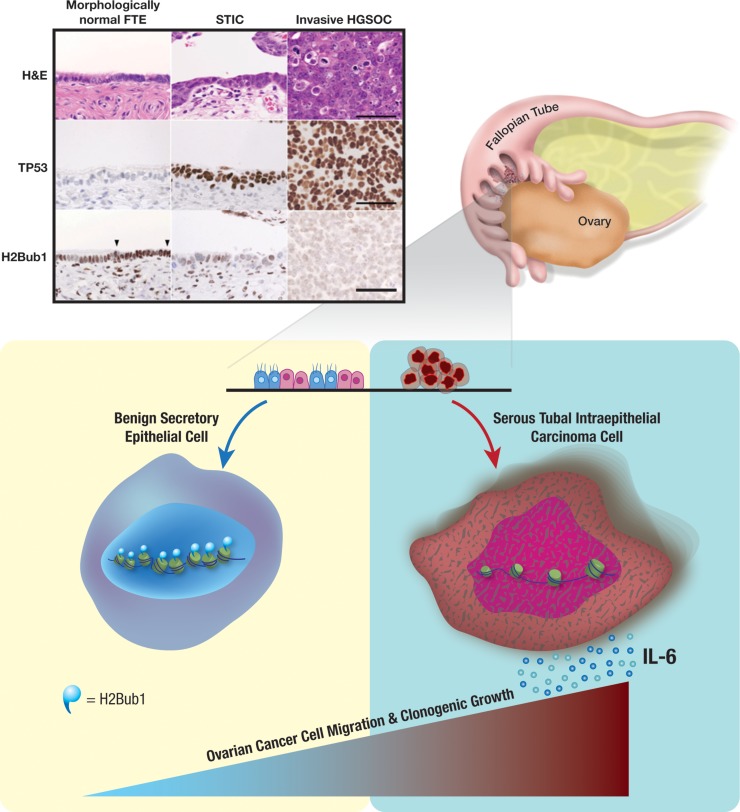
Loss of H2Bub1 in precursors of high-grade serous ovarian carcinoma leads to increased chromatin accessibility and expression of mediators that promote tumor cell migration. Upper left: Immunohistochemistry (IHC) staining of H2Bub1 in morphologically normal fallopian tube epithelium (FTE), Serous Tubal Intraepithelial Carcinoma (STIC), and high-grade serous ovarian carcinoma (HGSOC). The p53 IHC marks the carcinoma cells

However, DNA methylation may not be the only epigenetic alteration determining cell fate. We sought to investigate alternative post-translational modifications found in early aberrant FT cells that associate with HGSOC progression and identified a step-wise loss of histone H2B mono-ubiquitination (H2Bub1) at Lysine 120 (Figure 1) [8]. In order to characterize the functional implications of this epigenetic alteration, we modeled loss of H2Bub1 by knocking down its E3 ubiquitin ligase, RNF20, in FT cell lines. Depletion of RNF20 and consequently H2Bub1, led to an increase in FT cell migration, clonogenicity, and sphere formation. To understand how loss of this epigenetic mark triggers these malignant properties, we performed RNA-sequencing and Assay for Transposase-Accessible Chromatin using sequencing (ATAC-seq) analyses of these cells. Surprisingly, we found that loss of H2Bub1 allows for chromatin relaxation, promoting distinct euchromatic regions. The accessibility of these regions was associated with increased expression of immune signaling genes, including interleukin-6 (IL-6). IL-6 was shown to mediate the migratory phenotype of the FT cells and could be neutralized by anti-IL-6 antibodies, a therapeutic concept in clinical development. All of these results delineate an early epigenetic change that is seen in patients, and identify a mechanism that may be physiologically perpetuating disease progression towards HGSOC.

Gradual loss of H2Bub1 may explain how initiated, *TP53*-mutant FT cells undergo epigenetic and consequent genetic reprogramming that drive morphological transformation of the cells themselves, as well as alteration of the growing tumor microenvironment. For example, increased IL-6 cytokine release may sculpt a pro-inflammatory state around mutant precursors to allow migration without triggering immune surveillance pathways. Previous studies from Tarcic, et al. showed that prolonged inflammatory microenvironments stimulated by *RNF20* heterozygosity can encourage colon cancer development directly stemming from the epigenetic alterations induced by H2Bub1 loss [9]. IL-6 has also been specifically implicated in metastatic niche priming of the liver by primary pancreatic cancers [10]. Together, this crosstalk between cells secreting IL-6 and sites of secondary tumor formation provides a potential explanation for the gradual evolution from aberrant FT cell migration to eventual colonization of the ovary.

The ultimate goal of understanding how these epigenetic changes may be driving HGSOC tumorigenesis is to be able to therapeutically target active pathways. With all of the recent data emphasizing methylome reprogramming during the FT oncogenic switch, DNA methyltransferases (DNMTs) have become a promising drug target in ovarian cancers [11]. In terms of the emerging importance of H2Bub1 loss in HGSOC development, we seek to similarly approach ubiquitin epigenetics in FT cells. The E3 ligase, RNF20 provides a potential enzymatically active target in this pathway, but we believe that there are also relevant deubiquitinating enzymes (DUBs) that may be hyperactive and more easily targetable. As the field continues to progress, we aim to better characterize this and other early epigenetic alterations, providing insight into potential biomarkers of HGSOC development and novel therapeutic targets.
